# Supplementary material for: Comparative analysis of histone H3K4me3 modifications between blastocysts and somatic tissues in cattle
Source: Sci Rep. 2021 Apr 15;11:8253. doi: 10.1038/s41598-021-87683-0 (PMC8050253; doi:10.1038/s41598-021-87683-0)
Supplement: Supplementary file 1 — Supplementary Information 1. [file 41598_2021_87683_MOESM1_ESM.pdf]

Supplementary Information for

Comparative analysis of histone H3K4me3 modifications between blastocysts and somatic tissues in cattle

Mao Ishibashi, Shuntaro Ikeda*, Naojiro Minami

Laboratory of Reproductive Biology, Graduate School of Agriculture, Kyoto University, Kyoto 606-8502, Japan.

*Corresponding author

Email: ikeda.syuntaro.6u@kyoto-u.ac.jp

https://orcid.org/0000-0002-4939-2135

Supplementary figure legends and table titles

Figure S1. Correlation analysis of triplicates of ChIP-seq for H3K4me3 in bovine blastocysts. Scatterplots of three pairwise comparisons are shown with Pearson correlation coefficients. Ten kb bin size was used for drawing using deepTools (https://deeptools.readthedocs.io/en/develop/).

Figure S2. (a) H3K4me3 landscapes of the housekeeping genes *GAPDH* and *SDHA* derived from blastocyst, liver, and muscle ChIP-seq data. (b) Average profile plot of H3K4me3 signals around the TSSs of 165 meat production-related genes (left) and 20 imprinted genes (right), that harbor the modifications within ±3,000 bp of the TSSs both in blastocysts and muscle.

Figure S3. (a) Blastocyst- and (b) liver-specific H3K4me3 peaks around the TSSs of imprinted genes.

Figure S4. Comparison of the average profile plots of H3K4me3 signals around the genome-wide TSSs between the present and previous studies (1). *Replicate 1 of the published data was used for plotting.

Figure S5. H3K4me3 signals at *XIST* on chromosome X. (a) H3K4me3 signals in bovine unsexed cohort blastocysts (replicate 1 of the present study) and male somatic tissues. H3K4me3 peak was observed at the TSS region of *XIST* only in blastocysts. Liver, muscle, and brain (cerebral cortex) peaks were calculated from E-MTAB-2633 (bull4), GSM4800136, and GSM4800112, respectively. (b) H3K4me3 signals in human somatic tissues. Distinctive H3K4me3 peaks were observed at the TSS region of *XIST* only in female tissues. The human data were obtained from GSM537697 (female liver), GSM537709 (male liver), GSM621685 (female muscle), GSM621694 (male muscle), GSM806935 (female fetal brain), and GSM621457 (male fetal brain).

Table S1. Data generated by ChIP-seq analysis of bovine blastocysts using an anti-H3K4me3 antibody. (provided as .xlsx file)

Table S2. Top 10 significantly enriched GO terms derived from liver- and blastocyst-peaks, respectively, without sieving. (provided as .xlsx file)

Table S3. Top 10 significantly enriched GO terms derived from blastocyst-specific peaks relative to liver and muscle. (provided as .xlsx file)

Table S4. Meat production-related genes analyzed in this study. (provided as .xlsx file)

1. T. Org *et al.*, Genome-wide histone modification profiling of inner cell mass and trophectoderm of bovine blastocysts by RAT-ChIP. *PLoS One* **14**, e0225801 (2019).

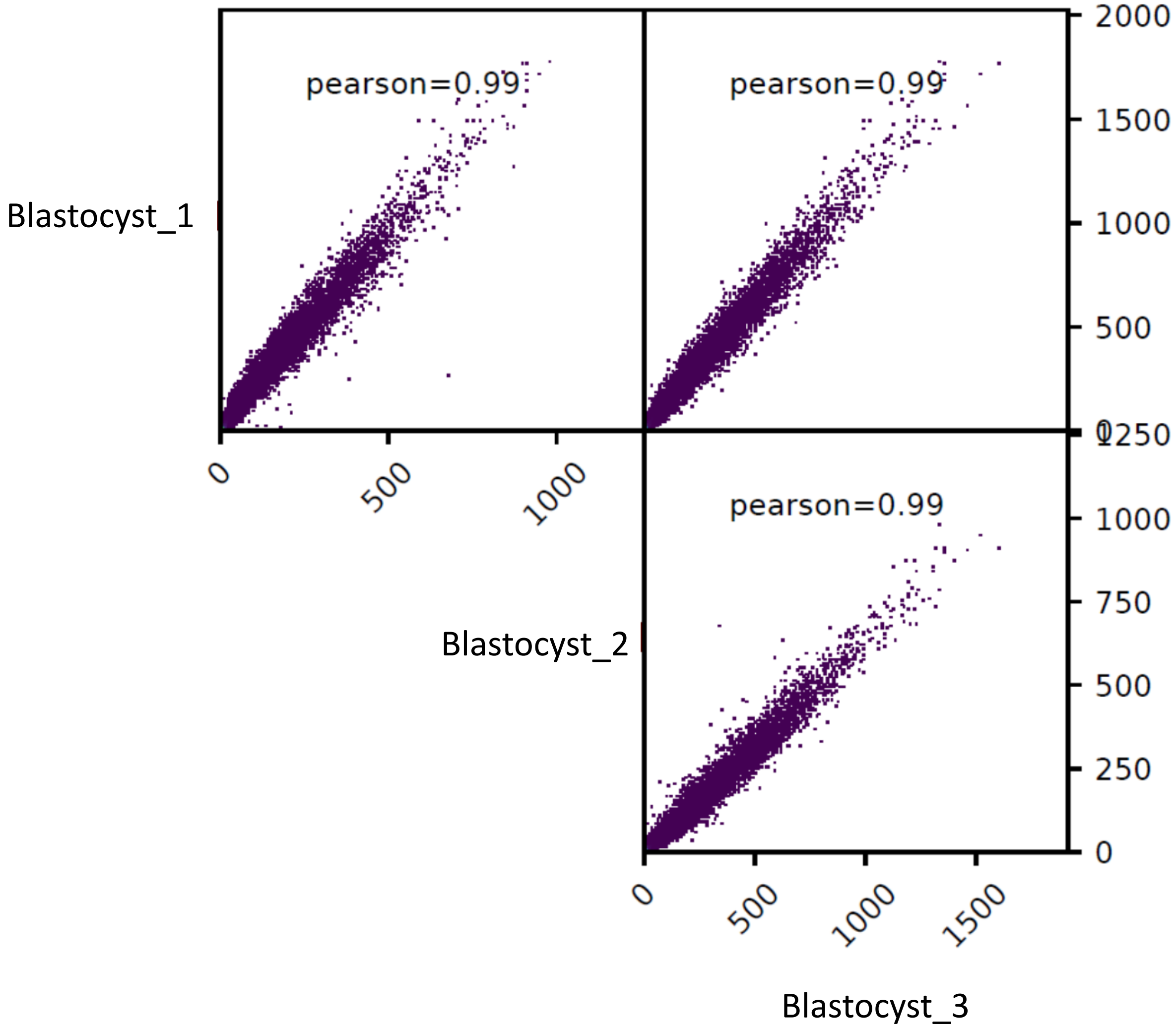

pearson=0.99
pearson=0.99
pearson=0.99
Blastocyst_1
Blastocyst_2
Blastocyst_3
2000
1500
1000
500
0
1250
1000
750
500
250
0
0
500
1000
0
500
1000
1500

Fig. S1

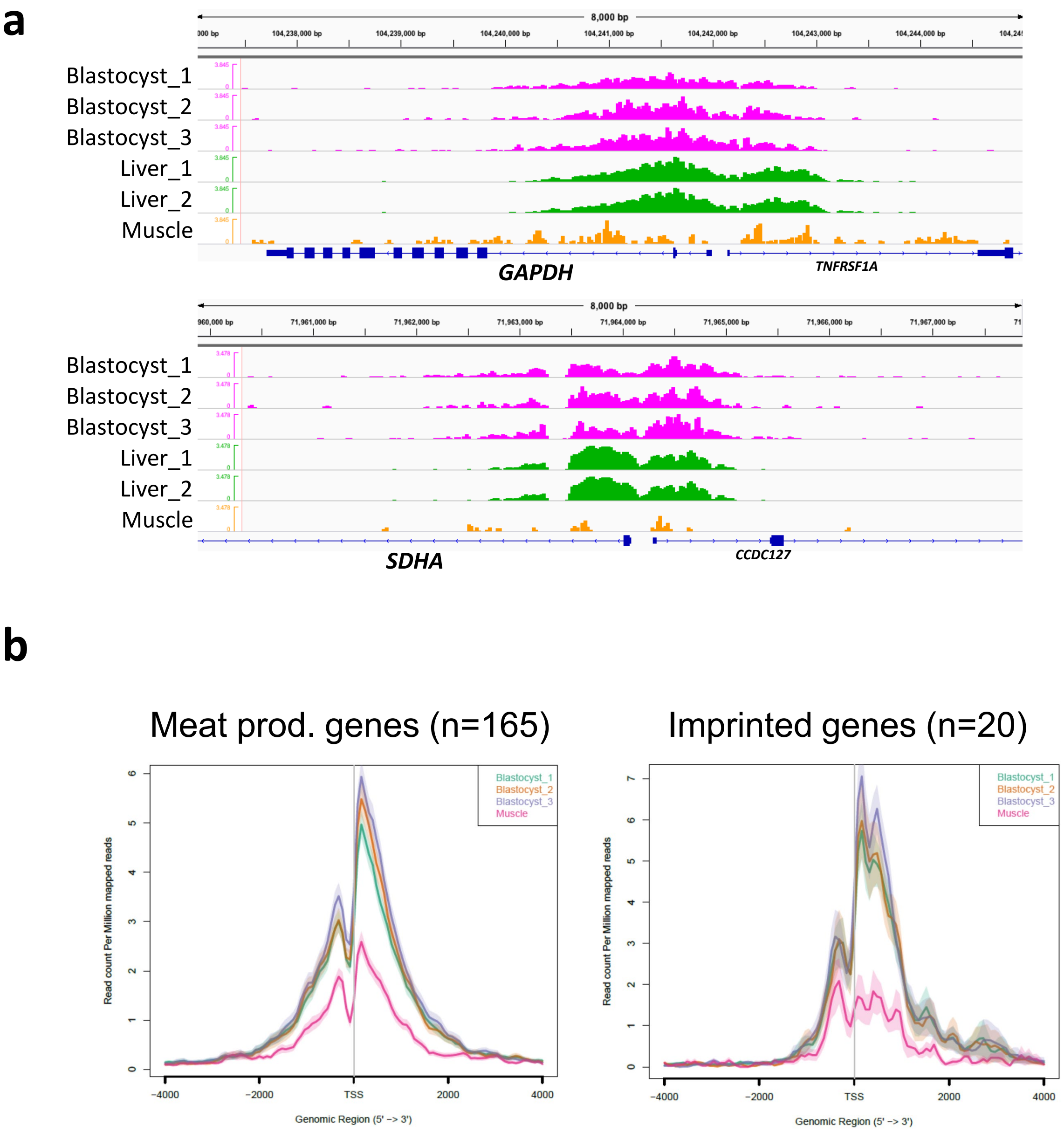
a
8,000 bp
Blastocyst_1
Blastocyst_2
Blastocyst_3
Liver_1
Liver_2
Muscle
GAPDH
TNFRSF1A
SDHA
CCDC127
b
Meat prod. genes (n=165)
Imprinted genes (n=20)
Blastocyst_1
Blastocyst_2
Blastocyst_3
Muscle
Read count Per Million mapped reads
Genomic Region (5' -> 3')
-4000
-2000
TSS
2000
4000

Fig. S2

a Blastocyst-specific

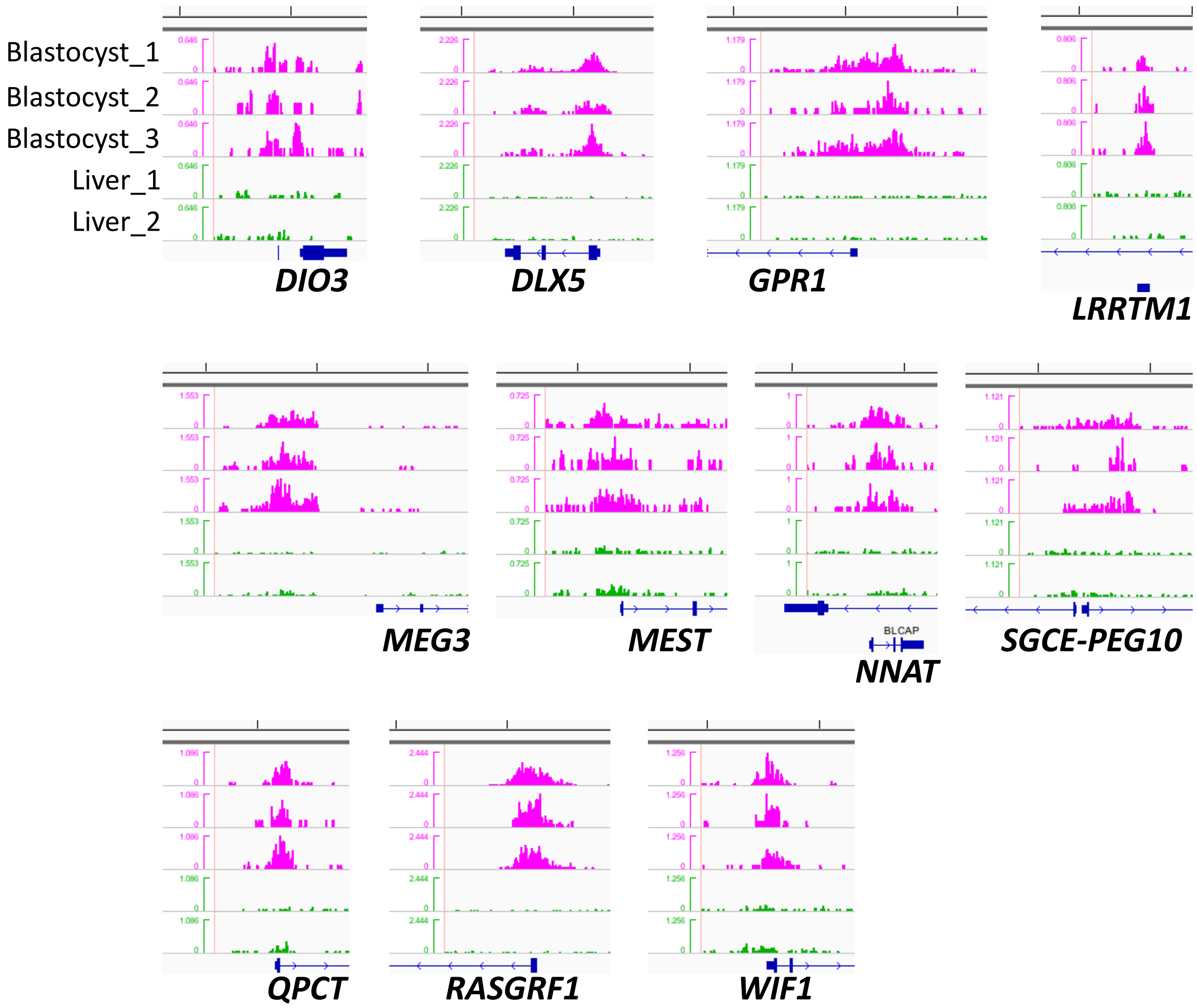

b Liver-specific

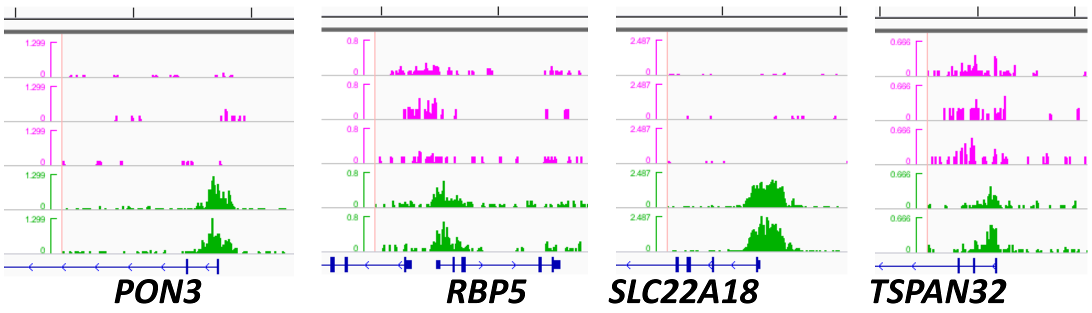

Fig. S3

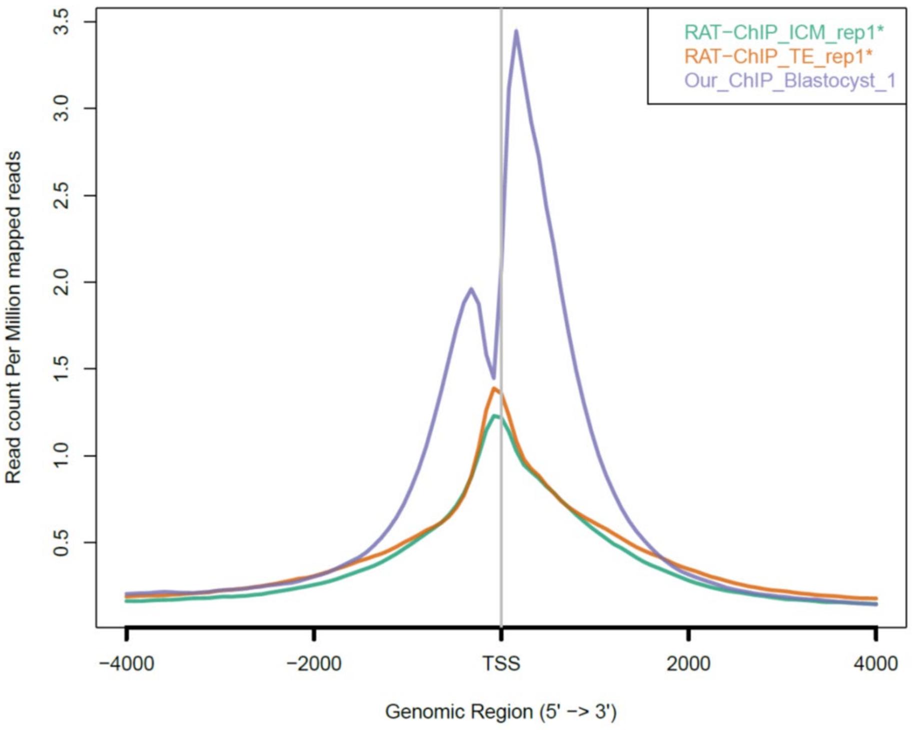
RAT−ChIP_ICM_rep1*
RAT−ChIP_TE_rep1*
Our_ChIP_Blastocyst_1
Read count Per Million mapped reads
3.5
3.0
2.5
2.0
1.5
1.0
0.5
−4000
−2000
TSS
2000
4000
Genomic Region (5' −> 3')

Fig. S4

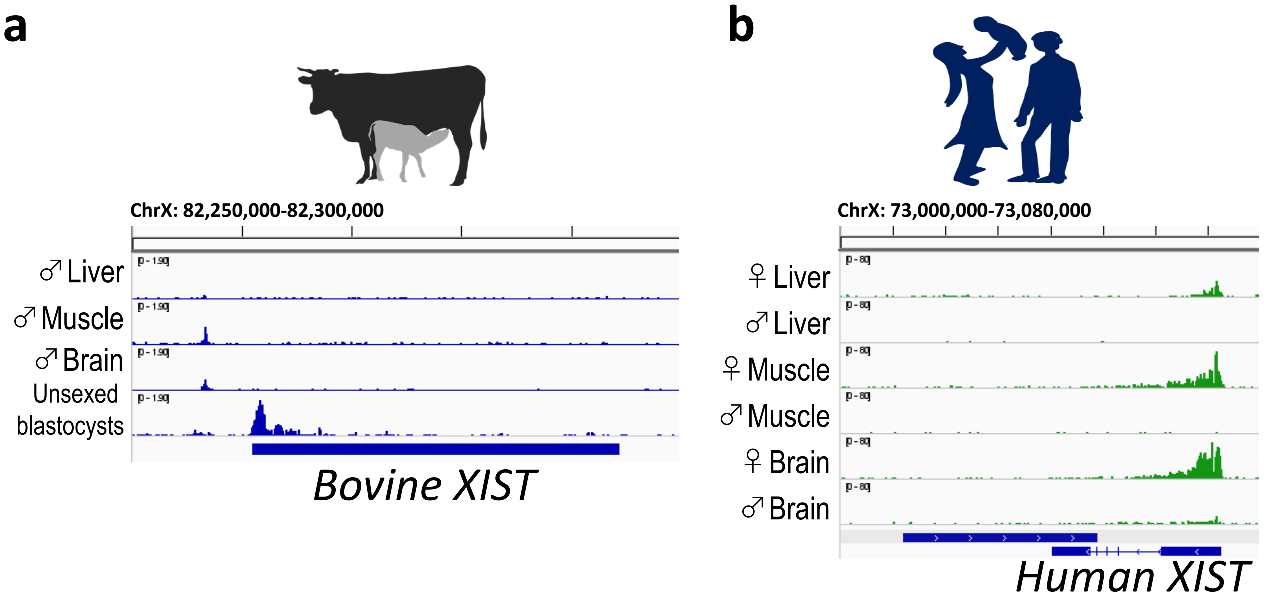
a
ChrX: 82,250,000-82,300,000
♂ Liver
♂ Muscle
♂ Brain
Unsexed blastocysts
Bovine XIST
b
ChrX: 73,000,000-73,080,000
♀ Liver
♂ Liver
♀ Muscle
♂ Muscle
♀ Brain
♂ Brain
Human XIST

Fig. S5
